# Supplementary figures and images for: Resting-State Co-activation Patterns as Promising Candidates for Prediction of Alzheimer’s Disease in Aged Mice
Source: Front Neural Circuits. 2021 Jan 22;14:612529. doi: 10.3389/fncir.2020.612529 (PMC7862346; doi:10.3389/fncir.2020.612529)

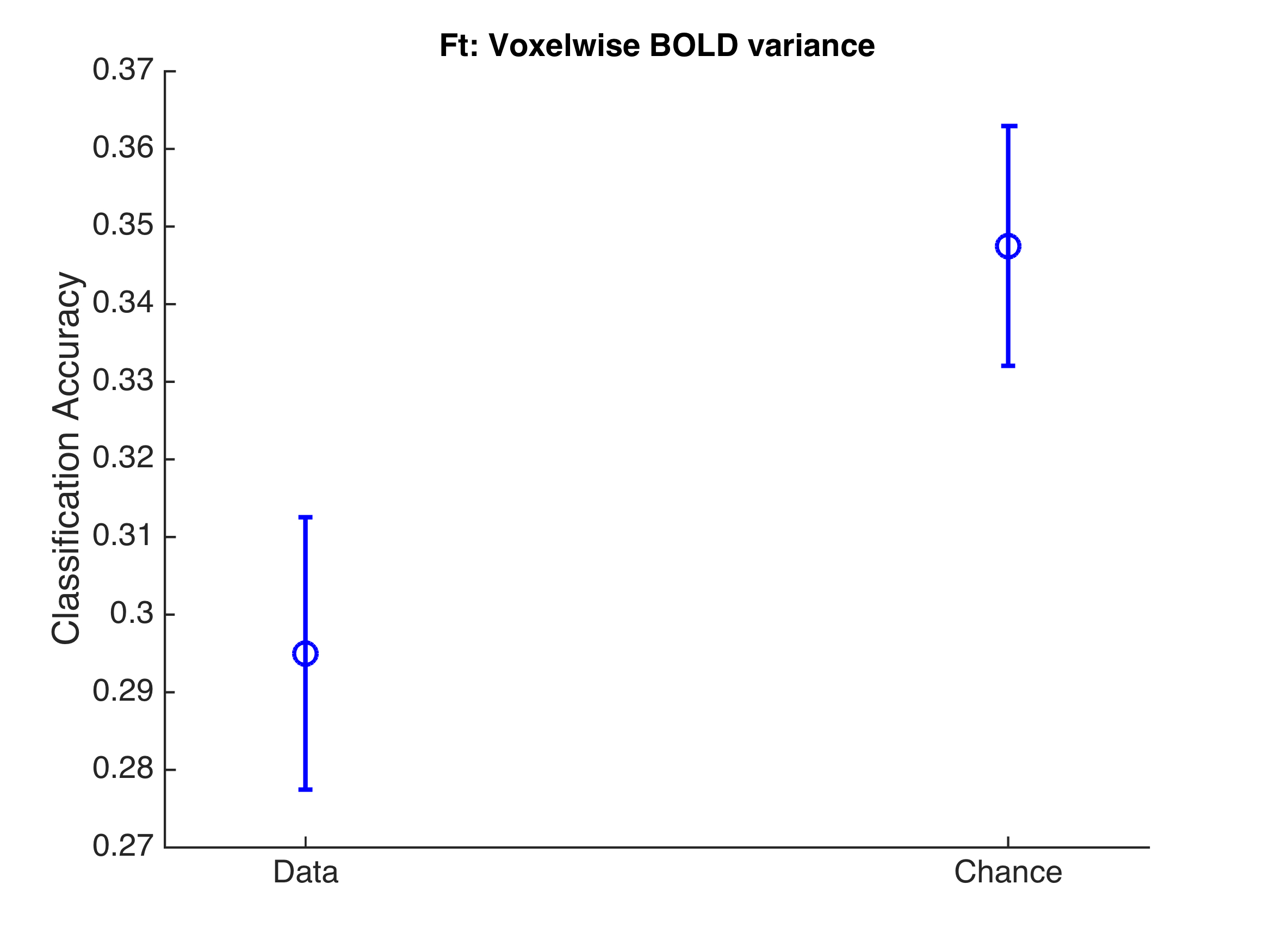

Supplement: SUPPLEMENTARY FIGURE 1 — Voxelwise BOLD variance does not distinguish TG from WT accurately. Here, we trained the classifier using the voxelwise variance of the BOLD signals (motion corrected, smoothed and filtered but without the z-score normalization) from 80% of subjects and tested on the remaining 20%. Mean classification accuracy, across 100 repetitions of random train-test splits was ~30% whereas the mean chance-level accuracy was ~35%. [file Image_1.jpg]
